# Supplementary material for: Enhancing CO2-Valorization Using Clostridium autoethanogenum for Sustainable Fuel and Chemicals Production
Source: Front Bioeng Biotechnol. 2020 Mar 27;8:204. doi: 10.3389/fbioe.2020.00204 (PMC7135887; doi:10.3389/fbioe.2020.00204)
Supplement: Supplementary file 2 [file Data_Sheet_1.PDF]

1 Materials and Methods

1.1 Data analysis

1.1.1 Analysis of flux data

Analysis of flux data from flux balance analysis (FBA) was performed to assist hypothesis formation. In relation to Table S7, flux data was normalized by CO_2 uptake (rxn05467_c0) before the differential change was calculated. Column headings indicate what flux was subtracted from one another. In relation to Table S8 & S9, and Figure S2, flux data was normalized by carbon uptake (i.e. CO uptake [rxn10480_c0] plus CO_2 uptake [rxn05467_c0]) CO_2 flux was included only if an uptake was present. Thus, for fermentations primarily fermenting CO, production of CO_2 from CO was not subtracted from carbon uptake. Relative fluxes were calculated by dividing flux ij by the respective (normalized) flux of $CO/CO_2/H_2$[1] – where i represents any fermentation dataset except for $CO/CO_2/H_2$[1], and j represents any reaction. Similarly, differential fluxes were calculated by taking flux ij and subtracting the respective (normalized) flux of $CO/CO_2/H_2$[1].

1.1.2 Gibb’s free energy balance

A Gibb’s free energy balance was conducted based on Liu et al. (2016). Free energy reactions from CO_2 to product ($\Delta G^0_{CO_2 \to product}$) were used to energetically describe each reaction. CO_2 was chosen as the base carbon molecule, as CO can readily be converted to CO_2 by cells. Volumetric free energy flow rates were calculated rather than amount of energy (i.e. kJ/L/day vs. J). Therefore, the $\Delta G^0_{CO_2 \to product}$ for each reaction was multiplied by its corresponding volumetric molar flow rate ($\dot{n}_{product}$ [mol/L/day]) from Table S6, to obtain a change in Gibb’s free energy flowrate for the overall system ($\Delta \dot{G}^0_{OR}$[kJ/L/day]). A biomass molecular weight of 24 g/mol was used to obtain $\dot{n}_{biomass}$ (Valgepea et al., 2017). $\Delta_r G^0_{CO_2 \to product}$ for biomass formation [1] was taken from Liu et al. (2016), while ethanol [2] and acetate formation [3] were found by reversing their respective combustion reactions, and combustion of H_2 [4] and CO [5] were used for their respective reactions. Aside from biomass, Chemistry Reference contained the appropriate ΔG^0_R information.

$$CO_2 + 0.24\,NH_3 + 0.525\,H_2O \rightleftharpoons CH_{1.77}O_{0.49}N_{0.24} + 1.02\,O_2 \qquad [1]$$
$$\Delta G^0_R = 479\,kJ/mol$$

$$2\,CO_2 + 3\,H_2O \rightleftharpoons CH_3CH_2OH + 3\,O_2 \qquad [2]$$
$$\Delta G^0_R = 1300\,kJ/mol$$

$$2\,CO_2 + 2\,H_2O \rightleftharpoons CH_3COOH + 2\,O_2 \qquad [3]$$
$$\Delta G^0_R = 873\,kJ/mol$$

$$H_2 + {}^1/_2\,O_2 \rightleftharpoons H_2O \qquad [4]$$
$$\Delta G^0_R = -230\,kJ/mol$$

$$CO + {}^1/_2\,O_2 \rightleftharpoons CO_2 \qquad [5]$$
$$\Delta G^0_R = -257\,kJ/mol$$

Typically, concentrations, not fluxes, are used for these calculations. However, absolute quantification of some intracellular metabolite concentrations are difficult to obtain (Lange et al., 2001 & Taymaz-Nikerel et al., 2009). To say definitively whether a reaction is spontaneous or not ($-\Delta G^0_R$ and ΔG^0_R respectively), one would have to know the intracellular concentrations. 0 kJ/mol is therefore the switch point between a

reaction being spontaneous and non-spontaneous. Importantly, time only affects the magnitude of the value, as it must be positive – thus, the rule holds when using rate values (it may be important to compare similar dilution rates though). We know that the system is negative ΔG because we see growth and/or conversion of substrates to products. Here, the standard conditions are assumed, meaning the calculations are not quantitative absolutely, but between comparable datasets results are quantitative relatively.

1.1.3 Titer conversion to specific production rate and retention factor correction

Using methods from Villadsen et al. (2011) (pg. 401), the titers from Mock et al. (2015) were converted to chemostat-equivalent specific production rates. Titers were 1.83, 6.3 and 7.48 g/L for biomass, ethanol and acetate respectively. Gas stripping of ethanol was found to be only 1% of total ethanol production here, therefore having minimal impact on the results reported for retentostat culture (not analyzed by Mock et al. [2015], based on $CO/CO_2/H_2$ cultures due to higher ethanol titers). Dilution rates were 0.5 and 4.9 day^{-1} for biomass and metabolites respectively. These were assumed to be growth rate (μ) and dilution rate (D), respectively, in the equation $\mu = D(1-\Omega)$, where $\Omega = 0.9$ was the retention factor. It was assumed that the metabolites from the bleed stream were retained, leading to a productivity increase factor of $(1-\Omega)^{-1}$. E.g. for ethanol the specific production rate was calculated by;

$$q_{EtOH} = (1-\Omega)\frac{D \times n_{EtOH}}{x} = 37.4\, mmol/gDCW/day$$

Where n_{EtOH} was the molar concentration of ethanol, x was the biomass concentration of the reactor, and q was the specific production rate of ethanol. When converted, Mock et al. (2015) observed lower specific productivities for retentostat CO_2/H_2 cultures of *C. autoethanogenum* (DSM 10061) compared to our chemostat fermentations with DSM 19630 (140 ± 10 vs 37, and 113 ± 9 vs 34 mmol/gDCW/day respectively), while the ethanol/acetate ratio was similar (1.2 vs 1.1 mol/mol respectively). However, the advantage of retentostat culture is volumetric production, hence their frequent use in CO_2 fermentation (Table 2). However, Mock et al.'s study focused on the biochemical reactions of gas fermentation, and unfortunately, a mass balance was not attempted. Thus, meaningful metabolic comparisons between CO_2/H_2 chemostats and retentostats are not possible yet; as both the strain of *C. autoethanogenum* and the culture conditions are different between Mock et al. and the data reported here.

2 Notes

2.1 Growth feedback loop and the mass transfer rate-dilution rate relationship

Non-equilibrium thermodynamics have been theorized to dictate metabolism and cell growth (Igamberdiev and Kleczkowski, 2009; Qian and Beard, 2005; Quéméner and Bouchez, 2014). Gas fermenting microbes create a mass transfer, non-equilibrium feedback loop, in that increasing biomass proportionally increases gas consumption – generating further increases in the mass transfer non-equilibrium driving force (Ma et al., 2005). Thus, continuous culture steady-states of gas fermenting microbes exemplify the principle of stable, non-equilibrium thermodynamics. Microbial growth intrinsically links mass transfer rate (MTR) and dilution rate (D) – design parameters known to be important for optimization of continuous gas fermentation performance (Klasson et al., 1991 & 1992). Furthermore, we have now explicitly shown that changes to MTRs (Valgepea et al., 2017a & 2018), and now D (Figure 2), lead to metabolic shifts. Substrate partial pressure, bubble size, and contact time are variables that are controllable and affect MTRs significantly (Bouaifi et al., 2001; Chen et al., 2018; Li et al., 2019). Changes to stirring speed and partial pressure caused a MTR difference between CO_2/H_2 and $CO/CO_2/H_2^{0.5}$. A ~5-fold increase in ethanol volumetric productivity was observed for $CO/CO_2/H_2^{0.5}$ compared to CO_2/H_2, while biomass concentration and acetate volumetric productivity observed a ~2-fold increase (Table 2). Yet, the specific flux through the WLP was not significantly affected, agreeing with results from Valgepea et al. (2017a). Varying results have been obtained with elevated partial pressures of H_2 and CO_2 (Table 2) (Demler and Weuster-Botz, 2011; Kantzow and Weuster-Botz, 2016; Oswald et al., 2018). However, the H_2:CO_2 ratios used in these studies were 2:1, therefore potentially leading to an H_2-limited fermentation. Here and by others (Table 2), H_2 to CO_2 consumption can be greater than 2:1. Thus, the hypothesis of bicarbonate acidification by Oswald et al. (2018) (also seen by Kantzow and Weuster-Botz [2016]), may only hold at this substrate feed ratio. This highlights an important difference between CO and CO_2 fermentations – the necessity of an energy substrate

(for CO_2) creates an imperative balance between carbon and redox metabolisms. Here, there was no observable improvements to MTRs compared to Valgepea et al. (2018) (shown by volumetric flux in Table S6). To improve MTRs outside of optimizing the MTR-D relationship or without decreasing specific productivities, it may be necessary to reduce any metabolic limitations (i.e. product inhibition) through methods like synergistic co-culture (Charubin and Papoutsakis, 2019; Richter et al., 2016a).

Increases in dilution rate and MTR led to the $CO/CO_2/H_2^{1}$ steady-state, indicating (when considering CO_2/H_2 vs $CO/CO_2/H_2^{0.5}$) that specific growth rate (result of D) was most likely the cause of the WLP specific productivity increase. Previously, different dilution rates were not reported for *C. autoethanogenum* chemostats, and there appears to be a proportional relationship between the WLP flux and D. The ethanol/acetate ratio of $CO/CO_2/H_2^{1}$ also decreased by close to half compared to $CO/CO_2/H_2^{0.5}$, and approached that of CO_2/H_2 fermentation (Figure 1). This suggests that metabolism was approaching a metabolic state similar CO_2/H_2 fermentation, and once again may be struggling to balance redox. One solution to this could be increasing CO supplementation to facilitate higher biomass and ethanol production (as seen for CO_2/H_2 vs $CO/CO_2/H_2^{0.5}$). Additionally, retentostat or multi-stage chemostat set-ups can reduce growth rate pressure while also reducing product inhibition (Table 2) (Mock et al., 2015; Molitor et al., 2019; Richter et al., 2013). This tends to increase the volumetric-, but reduce the specific-productivity, a trade-off that is generally desirable industrially. Furthermore, CO supplementation could increase volumetric productivities for CO_2/H_2 retentostats. However, the feasibility of attaining the same fold-increase in retentostat cultures is yet to be seen.Thus, further work towards optimization of these parameters may enhance not only the reactor performance, but also the metabolic performance.

2.2 Enzymes characterizations of interest

C. autoethanogenum possesses many vitally important enzymes. However, within the acetogen community, there is a diversity of these enzymes, and even more so for some present in the wider communities. We briefly discussed the Rnf complex diversity within acetogens, with more in-depth analysis by Bengelsdorf et al. (2018) and Poehlein et al. (2015). The following discusses other enzymes with notable importance and potential for further characterization.

2.2.1 CODH

Enzyme specific activity may play a role in the WLP increase. Mock et al. (2015) found that between CO and CO_2/H_2 cultures, the largest (~2-fold) significant activity increase was in carbon monoxide dehydrogenase (CODH). Clearly, CODH plays an important role in CO_2 fermentation, as it is reducing CO_2 instead of oxidizing CO (Figure 2) – an energetically less favorable reaction. Mock et al. (2015) also observed that of the two highly transcribed CODH genes (CAETHG_1620-21 & 3005), the split gene formed a complex with acetyl-CoA synthase (ACS). Thus, the complex also catalyzes the most energetically unfavorable WLP reaction – the production of acetyl-CoA (Norman et al., 2018). Knocking-out this complex was lethal to *C. autoethanogenum* DSM 10061 growing autotrophically (Liew et al., 2016a). Additionally, a CAETHG_3005 knockout (KO) strain showed striking growth rate improvements when fermenting CO_2/H_2 compared to the wild type. However, this KO was detrimental to CO fermentation, where they observed a significant decrease in culture performance. Subsequently, Liew et al. (2016a) hypothesized that the extra CODH may act as a competitor for CO_2. In CO-containing fermentations by Valgepea et al. (2018) (high- and low-BC), it appeared that an enzyme after CODH in the WLP was limiting flux (Figures 2 & S3). Here, however, the WLP flux doubled with CO supplementation. Plausibly, an increase in CODH/ACS complex activity caused this. The mechanism for such an activity increase is unknown. We hypothesize there are four plausible options: increased enzyme turnover (better thermodynamics); increased enzyme concentration (upregulation); a combination of the first two; or isozyme-substrate competition – as hypothesized by Liew et al. (2016a). Confirming a mechanism, could make it possible to engineer the complex and gain further CO_2 or CO fermentation improvements. One means of testing this mechanism is to compare CO-supplemented CO_2/H_2 fermentation by wild type and CAETHG_3005 KO *C. autoethanogenum* on a multi-omics level.

2.2.2 Hydrogenase

Interestingly, we observed higher specific uptake of H_2 in $CO/CO_2/H_2$ compared to CO/H_2. Comparatively, HytABCDE (leq000001) production of reduced ferredoxin appears to drive AOR and Rnf complex (leq000004 and M002 respectively) in $CO/CO_2/H_2$[1]. Moreover, Nfn complex (leq000002) utilizes NADPH from HytABCDE, also to produce reduced ferredoxin. Higher ATP production, uses reduced ferredoxin *via* Rnf complex, and assists excretion of acetate from the cell, reducing product inhibition of the WLP and acidification of the cell (Figure 2) (Valgepea et al., 2018). Considering CO is a known inhibitor of hydrogenases (Adams, 1990; Bennett et al., 2000; De Lacey et al., 2007; Lubitz et al., 2007; Peters et al., 2015) a lower CO concentration may facilitate the increase in H_2 utilization compared to CO/H_2 fermentation. This is especially true for redox metabolism as the hydrogenase associated with carbon metabolism, HytABCDE/Fdh (rxn08518_c0) here (Wang et al., 2013), was found to have enhanced CO-tolerating properties in *Acetobacterium woodii* (Ceccaldi et al., 2017). These hypotheses agree with further relative and differential analysis of fluxes (Figure 2, and Tables S8 & S9). When normalized by carbon uptake, the differential change in HytABCDE flux between CO-containing fermentations and $CO/CO_2/H_2$[1] is greater than that of HytABCDE/Fdh. Yet, there is also ongoing discussion about CO activation of hydrogenases (Evans et al., 2016; Lamle et al., 2005), and CO involvement in hydrogenase maturation (CO ligand synthesis) (Bürstel et al., 2016; Fontecilla-Camps et al., 2007; Lubitz et al., 2014; Pagnier et al., 2016). Thus, there is still much to understand about how these enzymes function (Wittkamp et al., 2018), and their diversity (Peters et al., 2015).

2.2.3 AOR

For acetogenic, and especially CO_2/H_2 fermentation, redox metabolism is vital. The importance of AOR (leq000004) for *C. autoethanogenum*'s autotrophic redox metabolism was identified by Marcellin et al. (2016), and further characterized -enzymatically by Mock et al. (2015), -isozymatically by Liew et al. (2017), and -metabolically by Valgepea et al. (2018). When fermenting CO_2/H_2, it is believed that AOR activity is essential for ethanol production (Richter et al., 2016b), and dictates phenotypic differences between acetogens, for example *A. woodii* and *C. autoethanogenum* (Table 2)(Bertsch and Müller, 2015; Mock et al., 2015). As the AOR provides a reduction in acidification that also results in a industrially more valuable product, optimisation of this pathway could lead to industrially relevant shifts in carbon distributions.

3 Supplementary Tables

Supplementary Tables S10-12 are provided in another supplementary file for larger tables.

TABLE S1 | Results summary

Gas	Gas Composition (Ar to 100%)	Gas flowrate mL/min	Stirrer Speed rpm	Biological replicates	Dilution rate day^{-1}	Biomass gDCW/L	±	Acetate g/L	±	Ethanol g/L	±
CO	60% CO	50	510	4	1	0.47	0.02	2.12	0.18	0.63	0.05
Syngas	50 % CO, 20% CO_2, 20% H_2	50	500	2	1	0.48	0.04	4.35	0.12	0.61	0.06
CO/H_2	15% CO, 45% H_2	50	650	4	1	0.46	0.04	0.69	0.07	4.46	0.41
$CO/CO_2/H_2^{1}$	2% CO, 23% CO_2, 65% H_2	30	1200	2	1	0.34	0.02	5.03	0.34	4.79	0.43
CO_2/H_2	23% CO_2, 67% H_2	32	500	3	0.5	0.18	0.02	2.51	0.42	2.36	0.25
$CO/CO_2/H_2^{0.5}$	2% CO, 23% CO_2, 65% H_2	30	800	2	0.5	0.54	0.01	5.97	0.98	9.69	0.39

TABLE S2 | Flux ratios (mol%)

Gas	Gas feed ratio				Gas flux ratio				Difference (Gas feed – Gas flux)				Ethanol/substrate ratio (+acetate)				
	CO_2/H_2	CO/H_2	$CO+CO_2/H_2$	CO/CO_2	CO_2/H_2	CO/H_2	$CO+CO_2/H_2$	CO/CO_2	CO_2/H_2	CO/H_2	$CO+CO_2/H_2$	CO/CO_2	EtOH /Ace	EtOH $/H_2$	EtOH /CO	EtOH $/CO_2$	EtOH/ $CO+CO_2$
CO	-	-	-	-	-	-	-	-1.33	-	-	-	-	0.41	-	0.06	-	0.06
Syngas	1.00	2.50	3.50	2.50	-0.45	1.49	1.05	-3.35	1.45	1.01	2.45	5.85	0.20	0.10	0.07	-	0.07
CO/H_2	-	0.33	0.33	-	-0.06	0.61	0.54	-9.49	-	-0.27	-0.21	-	8.51	0.27	0.45	-	0.45
$CO/CO_2/H_2^{1}$	0.35	0.03	0.38	0.09	0.36	0.03	0.40	0.09	-0.01	0.00	-0.01	-0.01	1.26	0.12	3.43	0.32	0.30
CO_2/H_2	0.34	-	0.34	-	0.41	-	0.41	-	-0.06	-	-0.06	-	1.24	0.12	-	0.31	0.31
$CO/CO_2/H_2^{0.5}$	0.35	0.03	0.38	0.09	0.34	0.02	0.37	0.07	0.01	0.01	0.02	0.02	2.15	0.13	5.46	0.37	0.34

The horizontal line through the middle of the table indicates where the data is from; above the line is data from (Valgepea et al., 2018), and below the line is novel data.

TABLE S3 | Flux values for Figure 1 and metabolic modelling constraints (mmol/gDCW/day)

Gas	Value	Acetate	Ethanol	CO_2	CO	H_2
CO	AV	76	31	-394	523	-14
	SD	5	1	6	12	1
	SD%	6%	4%	1%	2%	-5%
Syngas	AV	152	30	-135	451	302
	SD	8	0	2	17	13
	SD%	5%	0%	1%	4%	4%
CO/H_2	AV	26	217	-51	481	793
	SD	3	5	4	30	72
	SD%	12%	2%	9%	6%	9%
$CO/CO_2/H_2^1$	AV	243	306	944	89	2611
	SD	16	27	23	2	57
	SD%	7%	9%	2%	2%	2%
CO_2/H_2	AV	113	140	459	0	1130
	SD	9	12	77	0	158
	SD%	8%	8%	17%	0%	14%
$CO/CO_2/H_2^{0.5}$	AV	91	197	538	36	1562
	SD	15	8	20	4	24
	SD%	16%	4%	4%	11%	2%

For simulations, fluxes were converted to mmol/gDCW/h. All simulations used a cysteine consumption of 0.27 mmol/gDCW/h from Valgepea et al. (2017).

TABLE S4 | Product carbon balance values for Figure 1 (C-mol%)

Gas	Value	Biomass	CO_2	Acetate	Ethanol
CO	AV	6.4	60.7	23.5	9.5
	SD	0.1	1.0	1.2	0.2
	SD%	1%	2%	5%	3%
Syngas	AV	7.7	25.0	56.3	11.1
	SD	0.3	0.5	1.1	0.3
	SD%	4%	2%	2%	3%
CO/H_2	AV	7.2	8.9	8.4	75.5
	SD	0.1	0.8	0.2	0.9
	SD%	1%	9%	3%	1%
$CO/CO_2/H_2^1$	AV	3.6	-	42.5	53.8
	SD	0.1	-	0.3	0.4
	SD%	2%	-	1%	1%
CO_2/H_2	AV	3.9	-	41.7	54.5
	SD	0.2	-	2.6	2.6
	SD%	6%	-	6%	5%
$CO/CO_2/H_2^{0.5}$	AV	3.5	-	30.2	66.3
	SD	0.2	-	2.7	2.4
	SD%	7%	-	9%	4%

AV – average, SD – standard deviation, SD% - standard deviation as a percentage of average flux.

TABLE S5 | Results summary for high-biomass comparison

Gas	Gas Composition	Gas flowrate	Stirrer Speed	Biological replicates	Dilution rate	Biomass		Acetate		Ethanol	
	(Ar to 100%)	mL/min	rpm		day^{-1}	gDCW/L	±	g/L	±	g/L	±
CO	60% CO	50	665	4	1	1.43	0.08	6.28	0.43	3.88	0.15
Syngas	50 % CO, 20% CO_2, 20% H_2	50	650	2	1	1.36	0.06	7.86	0.14	5.43	0.33
CO/H_2	15% CO, 45% H_2	120	1000	4	1	1.45	0.04	3.84	0.33	11.55	0.41
CO/CO_2/H_2	2% CO, 23% CO_2, 65% H_2	30	1200	2	1	0.34	0.02	5.03	0.34	4.79	0.43

The horizontal line through the middle of the table indicates where the data is from; above the line is data from (Valgepea et al., 2018), and below the line is novel data.

TABLE S6 | Volumetric flux and free energy balance of fermentations

Reaction	ΔG_R^0, kJ/mol	Volumetric Productivity, mol/L/day								
		CO		Syngas		CO/H_2		CO/CO_2/H_2[1]	CO_2/H_2	CO/CO_2/H_2[0.5]
		LBC	HBC	LBC	HBC	LBC	HBC			
Biomass	479	0.020	0.060	0.020	0.057	0.019	0.060	0.014	0.004	0.011
Ethanol	1300	0.014	0.089	0.014	0.123	0.099	0.274	0.105	0.026	0.107
Acetate	873	0.036	0.107	0.071	0.133	0.012	0.066	0.084	0.021	0.050
Hydrogen	-230	0	0	0.146	0.387	0.363	1.026	0.900	0.209	0.849
CO	-257	0.245	1.060	0.219	0.997	0.220	0.713	0.031	0	0.020
CO_2	0	-0.185	-0.727	-0.065	-0.410	-0.023	-0.152	0.326	0.084	0.293
**$\Delta \dot{G}_{OR}$, kJ/mol/day**		**-3.6**	**-31.1**	**0.6**	**-42.2**	**8.5**	**23.6**	**2.0**	**5.4**	**-12.3**

Fermentations from Valgepea et al. (2018) and this report. Biomass Gibb's free energy of formation was obtained from Liu et al. (2016). All other free energy values were obtained from (Chemistry Reference), see Data Analysis – 1.1.2 Gibb's free energy balance for details. LBC – low biomass concentration, HBC – high biomass concentration.

TABLE S7 | Metabolic highlights between CO_2-fermenting cultures

Reaction	Reaction Flux mmol/gDCW/day			CO_2-Normalised Differential Flux mol/mol CO_2		
	$CO/CO_2/H_2^1$	CO_2/H_2	$CO/CO_2/H_2^{0.5}$	$CO/CO_2/H_2^1$ - CO_2/H_2	$CO/CO_2/H_2^{0.5}$ - CO_2/H_2	1-0.5
**Exchange**						
CO_2	42.64	21.00	22.43			
CO	3.71	0.00	1.51	↗ 0.09	↗ 0.07	→ 0.02
H_2	114.81	54.21	63.13	↑ 0.11	↑ 0.23	↓ -0.12
Acetate	10.13	4.69	3.80	→ 0.01	↘ -0.05	↗ 0.07
Ethanol	12.56	5.83	8.06	→ 0.02	↗ 0.08	↘ -0.06
**Central Metabolism**						
CODH/ACS (Fdred)	-19.35	-10.52	-10.46	→ 0.05	→ 0.03	→ 0.01
Tetrahydrofolate ligase (ATP)	-23.09	-10.54	-11.98	→ -0.04	→ -0.03	→ 0.00
Methylene-THF oxidoreductase (NADPH)	-23.08	-10.53	-11.97	→ -0.04	→ -0.03	→ 0.00
Methylene-THF reductase (Fdred)	23.07	10.53	11.97	→ 0.04	→ 0.03	→ 0.00
Acetyl-P Transferase (ATP)	22.64	10.50	11.84	→ 0.03	→ 0.03	→ 0.00
AOR (Fdred)	-12.54	-5.82	-8.04	→ -0.02	↘ -0.08	↗ 0.06
Ethanol:NAD+ oxidoreductase (NADH)	-12.56	-5.83	-8.06	→ -0.02	↘ -0.08	↗ 0.06
HytA-E (Fdred)	45.86	21.84	25.57	→ 0.04	↑ 0.10	↘ -0.06
Nfn Complex (Fdred)	11.03	5.44	6.53	→ 0.00	→ 0.03	→ -0.03
Rnf Complex (Fdred)	-47.79	-21.51	-25.51	↘ -0.10	↓ -0.11	→ 0.02
ATPase (ATP)	18.31	8.22	9.87	→ 0.04	→ 0.05	→ -0.01
**Other**						
PFOR (pyruvate)	0.27	0.04	0.05	→ 0.00	→ 0.00	→ 0.00
Total specific ATP production rate (ATP)	40.96	18.72	21.71	↗ 0.07	↗ 0.08	→ 0.00
H2O (H2O excretion)	-15.55	-2.51	-1.94	→ 0.02	↗ 0.09	↘ -0.07
NGAM (ATP consumption)	5.40	4.03	3.10	→ 0.03	→ 0.04	→ 0.00

Average specific fluxes were used for all comparisons. “Exchange” and “Central Metabolism” reactions match to Figure S4. See Flux data analysis for calculation details. Metabolites in brackets show forward direction of reaction (i.e. Rnf complex consumes reduced ferredoxin). Arrow scales are: green-up >= 0.1, yellow-diagonal-up >= 0.05, yellow-sideways >= -0.05, yellow-diagonal-down >= -0.1, red-down < -0.1. See Figure S1 for full metabolite and enzyme names and Figure S2 for full enzyme names and reaction IDs.

TABLE S8 | Highlights of comparative flux analysis

Reaction	Flux, mmol/gDCW/h									Differential flux change compared to $CO/CO_2/H_2^1$, mmol/gDCW/h							
	a	b	c	d	e	f	g	h	i	a	b	c	d	e	f	h	i
ATP:acetate phosphotransferase	4.42	5.64	7.52	7.80	9.95	9.75	22.64	10.50	11.84	-18.22	-17.00	-15.12	-14.84	-12.69	-12.90	-12.14	-10.80
Proton transport	5.80	7.91	13.49	12.35	10.54	11.51	32.19	14.54	15.09	-26.39	-24.28	-18.70	-19.83	-21.65	-20.68	-17.65	-17.09
Methylene-THF reductase	4.88	6.39	7.96	8.54	10.39	10.35	23.07	10.53	11.97	-18.19	-16.67	-15.11	-14.53	-12.67	-12.72	-12.54	-11.10
Formate:THF ligase	4.91	6.42	7.99	8.57	10.42	10.37	23.09	10.54	11.98	-18.19	-16.67	-15.11	-14.53	-12.67	-12.72	-12.55	-11.11
5,10-Methenyl-THF 5-hydrolase	-4.89	-6.40	-7.97	-8.55	-10.40	-10.36	-23.08	-10.53	-11.97	18.19	16.67	15.11	14.53	12.67	12.72	12.54	11.11
ATP synthase	7.45	9.50	8.33	10.39	11.92	11.91	18.31	8.22	9.87	-10.87	-8.82	-9.98	-7.92	-6.40	-6.40	-10.10	-8.45
5,10-methylene-THF oxidoreductase	-4.89	-6.40	-7.97	-8.55	-10.40	-10.36	-23.08	-10.53	-11.97	18.19	16.67	15.11	14.53	12.67	12.72	12.54	11.11
Ethanol:NAD+ oxidoreductase	1.29	2.58	1.24	3.78	8.99	7.90	12.56	5.83	8.06	-11.27	-9.98	-11.32	-8.78	-3.57	-4.66	-6.73	-4.51
Acetyl-CoA acetyltransferase	4.42	5.64	7.52	7.80	9.95	9.75	22.64	10.50	11.84	-18.22	-17.00	-15.12	-14.84	-12.69	-12.90	-12.14	-10.80
NGAM	3.03	5.55	5.96	7.72	9.56	9.34	15.86	7.17	8.63	-12.83	-10.31	-9.90	-8.13	-6.29	-6.51	-8.69	-7.23
H2O transport	11.65	15.79	3.92	8.44	-7.53	-4.27	-56.04	-27.12	-30.97	67.69	71.83	59.96	64.48	48.51	51.78	28.92	25.07
CO2 transport	-16.42	-21.12	-5.61	-12.57	-2.11	-4.39	42.64	21.00	22.43	-59.07	-63.76	-48.25	-55.21	-44.75	-47.03	-21.65	-20.22
CO transport	-26.44	-34.32	-21.91	-30.57	-23.19	-25.54	-3.72	0.00	-1.51	-22.72	-30.60	-18.19	-26.85	-19.48	-21.83	3.72	2.21
CO dehydrogenase	21.57	27.93	13.95	22.03	12.80	15.20	-19.35	-10.52	-10.46	40.91	47.28	33.30	41.38	32.15	34.55	8.82	8.89
CODH/ACS	4.87	6.39	7.95	8.53	10.39	10.34	23.06	10.52	11.96	-18.19	-16.67	-15.11	-14.53	-12.67	-12.72	-12.54	-11.10
5-Methyl-THF methyltransferase	4.87	6.39	7.95	8.53	10.39	10.34	23.06	10.52	11.96	-18.19	-16.67	-15.11	-14.53	-12.67	-12.72	-12.54	-11.10
H2 transport	0.58	0.49	-13.47	-12.99	-37.39	-32.84	-114.81	-54.21	-63.13	115.38	115.30	101.34	101.82	77.41	81.97	60.59	51.68
Ethanol transport	1.29	2.58	1.24	3.78	8.99	7.90	12.56	5.83	8.06	-11.27	-9.98	-11.32	-8.78	-3.57	-4.66	-6.73	-4.51
Rnf complex	16.64	21.63	20.28	24.70	28.68	28.63	47.79	21.51	25.51	-31.15	-26.16	-27.51	-23.09	-19.11	-19.16	-26.28	-22.27
Acetate transport	3.18	3.11	6.32	4.06	1.00	1.89	10.13	4.69	3.80	-6.95	-7.02	-3.81	-6.07	-9.12	-8.24	-5.43	-6.32
HytABCDE	-0.29	-0.25	2.72	1.98	13.48	11.23	45.86	21.84	25.57	-46.14	-46.10	-43.13	-43.88	-32.37	-34.63	-24.02	-20.28
Nfn complex	5.48	6.08	2.84	3.50	-1.27	-0.18	-11.03	-5.44	-6.53	16.51	17.11	13.86	14.52	9.76	10.84	5.58	4.50
Formate hydrogen-lyase	0.00	0.00	8.02	9.02	10.43	10.37	23.09	10.54	11.98	-23.09	-23.09	-15.07	-14.07	-12.67	-12.72	-12.55	-11.11
AOR	1.27	2.56	1.22	3.76	8.97	7.88	12.54	5.82	8.04	-11.27	-9.98	-11.32	-8.78	-3.57	-4.66	-6.72	-4.49
PFOR	-0.31	-0.60	-0.29	-0.59	-0.30	-0.45	-0.27	-0.04	-0.05	-0.04	-0.33	-0.02	-0.32	-0.02	-0.17	0.32	0.22

TABLE S9 | Highlights of comparative flux analysis normalised by carbon uptake

Reaction	Absolute-relative flux change normalised by carbon uptake and compared to $CO/CO_2/H_2^1$, mol/mol								Differential flux change normalised by carbon uptake and compared to $CO/CO_2/H_2^1$, mol/mol							
	a	b	c	d	e	f	h	i	a	b	c	d	e	f	h	i
ATP:acetate phosphotransferase	0.34	0.34	0.70	0.52	0.88	0.78	1.02	1.01	0.32	0.32	0.15	0.23	0.06	0.11	-0.01	-0.01
Proton transport	0.32	0.33	0.89	0.58	0.65	0.65	1.00	0.91	0.47	0.46	0.08	0.29	0.24	0.24	0.00	0.06
Methylene-THF reductase	0.37	0.37	0.73	0.56	0.90	0.81	1.01	1.00	0.31	0.31	0.13	0.22	0.05	0.09	0.00	0.00
Formate:THF ligase	0.37	0.38	0.73	0.56	0.90	0.82	1.01	1.00	0.31	0.31	0.13	0.22	0.05	0.09	0.00	0.00
5,10-Methenyl-THF 5-hydrolase	0.37	0.37	0.73	0.56	0.90	0.81	1.01	1.00	-0.31	-0.31	-0.13	-0.22	-0.05	-0.09	0.00	0.00
ATP synthase	0.71	0.70	0.96	0.86	1.30	1.18	0.99	1.04	0.11	0.12	0.01	0.05	-0.12	-0.07	0.00	-0.02
5,10-methylene-THF oxidoreductase	0.37	0.37	0.73	0.56	0.90	0.81	1.01	1.00	-0.31	-0.31	-0.13	-0.22	-0.05	-0.09	0.00	0.00
Ethanol:NAD+ oxidoreductase	0.18	0.28	0.21	0.46	1.43	1.14	1.02	1.24	0.22	0.20	0.21	0.15	-0.12	-0.04	-0.01	-0.07
Acetyl-CoA acetyltransferase	0.34	0.34	0.70	0.52	0.88	0.78	1.02	1.01	0.32	0.32	0.15	0.23	0.06	0.11	-0.01	-0.01
NGAM	0.34	0.47	0.79	0.74	1.21	1.07	1.00	1.05	0.23	0.18	0.07	0.09	-0.07	-0.02	0.00	-0.02
H2O transport	0.36	0.38	0.15	0.23	0.27	0.14	1.07	1.07	-1.65	-1.67	-1.39	-1.49	-0.88	-1.04	0.08	0.09
CO2 transport	0.68	0.67	0.28	0.45	0.10	0.19	1.09	1.02	1.54	1.54	1.18	1.33	1.01	1.09	-0.08	-0.02
CO transport	12.48	12.48	12.48	12.48	12.48	12.48	0.00	0.78	0.92	0.92	0.92	0.92	0.92	0.92	-0.08	-0.02
CO dehydrogenase	1.95	1.95	1.53	1.73	1.32	1.43	1.20	1.05	-1.23	-1.23	-1.05	-1.14	-0.97	-1.01	0.08	0.02
CODH/ACS	0.37	0.37	0.73	0.56	0.90	0.81	1.01	1.00	0.31	0.31	0.13	0.22	0.05	0.09	0.00	0.00
5-Methyl-THF methyltransferase	0.37	0.37	0.73	0.56	0.90	0.81	1.01	1.00	0.31	0.31	0.13	0.22	0.05	0.09	0.00	0.00
H2 transport	0.01	0.01	0.25	0.17	0.65	0.52	1.04	1.07	-2.50	-2.49	-1.86	-2.05	-0.86	-1.19	0.11	0.16
Ethanol transport	0.18	0.28	0.21	0.46	1.43	1.14	1.02	1.24	0.22	0.20	0.21	0.15	-0.12	-0.04	-0.01	-0.07
Rnf complex	0.61	0.61	0.90	0.78	1.20	1.09	0.99	1.03	0.40	0.40	0.10	0.22	-0.21	-0.09	0.01	-0.04
Acetate transport	0.55	0.41	1.32	0.61	0.20	0.34	1.02	0.73	0.10	0.13	-0.07	0.09	0.18	0.14	-0.01	0.06
HytABCDE	0.01	0.01	0.13	0.07	0.59	0.44	1.05	1.08	1.00	1.00	0.86	0.92	0.41	0.55	-0.05	-0.08
Nfn complex	0.87	0.75	0.54	0.48	0.23	0.03	1.09	1.15	-0.45	-0.42	-0.37	-0.35	-0.18	-0.23	0.02	0.03
Formate hydrogen-lyase	0.00	0.00	0.74	0.59	0.90	0.82	1.01	1.00	0.50	0.50	0.13	0.20	0.05	0.09	0.00	0.00
AOR	0.18	0.28	0.21	0.45	1.43	1.14	1.02	1.24	0.22	0.20	0.21	0.15	-0.12	-0.04	-0.01	-0.07
PFOR	1.99	2.97	2.26	3.27	2.15	2.96	0.35	0.37	0.01	0.01	0.01	0.01	0.01	0.01	-0.01	0.00

Average specific fluxes were used for all comparisons. For full analysis see Figure S2. Figure S2 also contains full description and ID for noted reactions. a & b – CO low & high biomass, c & d syngas low & high biomass, e & f – CO/H_2 low & high biomass, g – $CO/CO_2/H_2^1$, h – CO_2/H_2, i – $CO/CO_2/H_2^{0.5}$. Division and subtraction by the corresponding flux of $CO/CO_2/H_2^1$, respectively, produced the relative and differential analysis. The absolute-relative change was used here (compared to non-absolute in Figure S2) to show change as an increase (green), decrease (red), and no change (black) in flux magnitude. Scales (red to green): (top left) -30 to 30, (top right) -50 to 50, (bottom left) 0 to 2, (bottom right) -1 to 1. Positive/negative values do not implicitly lead to higher/lower activity, respectively. These comparisons were used only as an indication of where change was occurring. To ascertain increase/decrease effect precisely see Supplementary Materials Table S11 for flux data.

4 Supplementary Figures

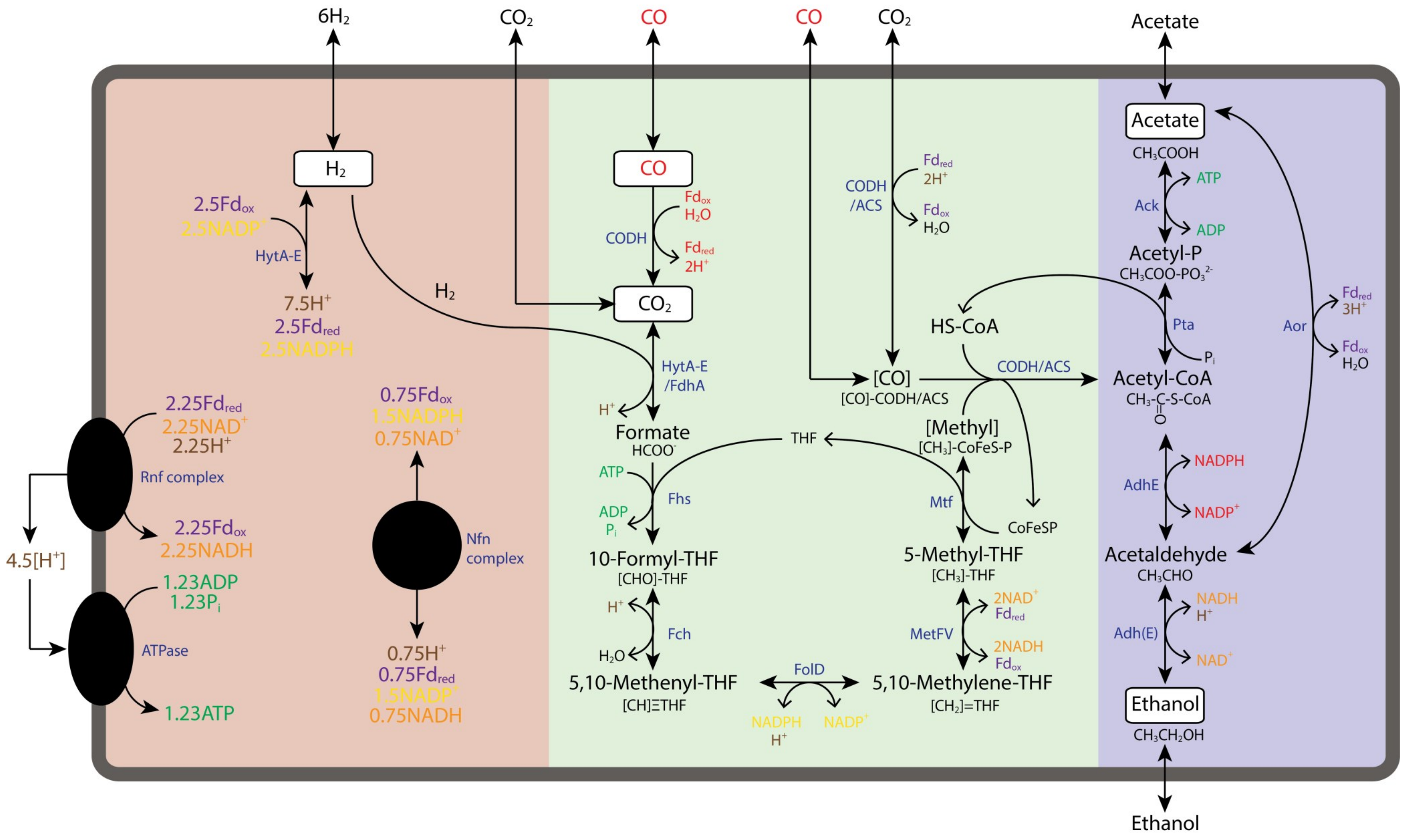

6H2
CO2
CO
CO
CO2
Acetate
H2
CO
CO2
2.5Fdox
2.5NADP+
HytA-E
7.5H+
2.5Fdred
2.5NADPH
H2
CODH
Fdox
H2O
Fdred
2H+
CODH
/ACS
Fdred
2H+
Fdox
H2O
Acetate
CH3COOH
Ack
ATP
ADP
Acetyl-P
CH3COO-PO3 2-
Pta
Pi
HS-CoA
CODH/ACS
Acetyl-CoA
CH3-C-S-CoA
O
Aor
Fdred
3H+
Fdox
H2O
HytA-E
/FdhA
H+
Formate
HCOO-
[CO]
[CO]-CODH/ACS
[Methyl]
[CH3]-CoFeS-P
THF
2.25Fdred
2.25NAD+
2.25H+
Rnf complex
2.25Fdox
2.25NADH
4.5[H+]
1.23ADP
1.23Pi
ATPase
1.23ATP
0.75Fdox
1.5NADPH
0.75NAD+
Nfn
complex
0.75H+
0.75Fdred
1.5NADP+
0.75NADH
ATP
Fhs
ADP
Pi
10-Formyl-THF
[CHO]-THF
H+
Fch
H2O
5,10-Methenyl-THF
[CH]≡THF
FolD
NADPH
H+
NADP+
Mtf
CoFeSP
5-Methyl-THF
[CH3]-THF
MetFV
2NAD+
Fdred
2NADH
Fdox
5,10-Methylene-THF
[CH2]=THF
AdhE
NADPH
NADP+
Acetaldehyde
CH3CHO
Adh(E)
NADH
H+
NAD+
Ethanol
CH3CH2OH
Ethanol

FIGURE S1 | The theoretical central metabolic pathways of *Clostridium autoethanogenum* when fermenting CO_2/H_2 to ethanol, based on schemes from Mock et al. (2015). Here, ferredoxin was assumed to be the electron acceptor of the reaction catalysed by MetFV and ethanol production was assumed to occur via the AOR. The overall reaction was $2CO_2 + 6H_2 + 1.23ADP + 1.23P_i \rightarrow CH_3CH_2OH + 3H_2O + 1.23ATP$. Reactions involving free CO and the reaction facilitated by AdhE (acetyl-CoA to acetaldehyde) are not active. Arrows show the reaction direction. **Background**: red – energetic/redox pathways, green – WLP, blue – product pathways. **Writing**: reaction not present (red), Fd_{ox} and Fd_{red} - oxidised and reduced ferredoxin (purple), NADH and NAD^+ – protonated and deprotonated nicotinamide adenine dinucleotide (orange), NADPH and $NADP^+$ - protonated and deprotonated NAD phosphate (yellow), ATP and ADP + P_i – adenosine triphosphate and precursors (green), H^+ – protons (brown), THF – tetrahydrofolate, **enzymes** (blue): HytA-E – electron bifurcating, NADP - and ferredoxin-dependent [FeFe]-hydrogenase, HytA-E/FdhA – HytA-E in complex with selenium- and tungsten-dependent formate dehydrogenase, CODH – carbon monoxide dehydrogenase, CODH/ACS – CODH complex with acetyl CoA synthase, Rnf – Fd_{red}:NAD^+ oxidoreductase, Nfn – ferredoxin-dependent transhydrogenase, ATPase – ATP synthase, Fhs – formate:THF ligase, Fch – 5,10-methenyl-THF 5-hydrolase, FolD – methylene-THF dehydrogenase, MetFV – methylene-THF reductase, Mtf - methyl-THF:CoFeSP transferase, Pta – phosphotransacetylase, Ack – acetate kinase, AdhE - bifunctional alcohol dehydrogenase/acetaldehyde dehydrogenase, Adh - alcohol dehydrogenase, Aor - acetylaldehyde:ferredoxin oxidoreductase.

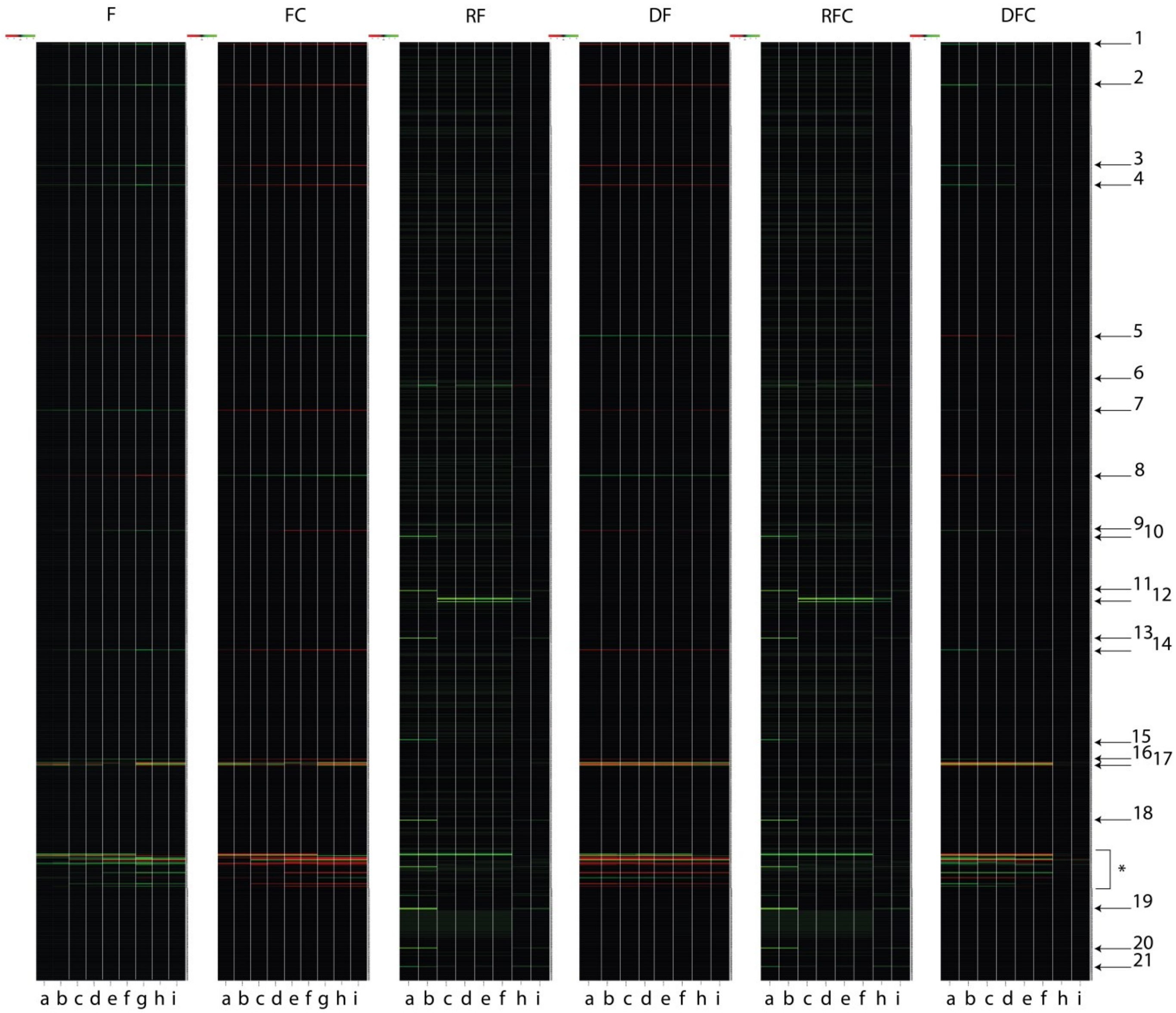

FIGURE S2 | Initial comparative flux analysis of entire FBA datasets with heatmaps. All fluxes are an average of the respective steady-state biological replicates, with further analysis also completed using these averages. F – flux, FC – flux normalised by carbon uptake, RF – relative flux, DF – differential flux, RFC – relative flux normalised by carbon uptake, DFC – differential flux normalised by carbon uptake. a & b – CO low & high biomass, c & d – syngas low & high biomass, e & f – CO/H_2 low & high biomass, g – $CO/CO_2/H_2^1$, h – CO_2/H_2, i – $CO/CO_2/H_2^{0.5}$. Division and subtraction by the corresponding flux of $CO/CO_2/H_2^1$ produced the relative and differential analysis, respectively. Colour scales are roughly (red to green respectively): -100 to 100 for F & DF, -2 to 2 for FC & DFC, -25 to 25 RF, and -50 to 50 for RFC. Results from Valgepea et al. (2018) are also displayed, the conditions of these fermentations are summarized in Table S1 & S7. Carbon uptake was a negative value, hence the directionality switch (e.g. F to FC). Reactions are displayed in the same order as in Supplementary Materials Table S11. See Tables S8 & S9 for highlights of the heatmap. Heatmaps were produced using Heatmapper (Babicki et al., 2016).

Reactions noted in the heatmap: 1 – acetate phosphotransferase (rxn00225_c0); 2 – proton transport (cpd00067_ext_b); 3 – NADH-dependent, electron-bifurcating, methylene-THF reductase (rxn00910_c0); 4 – formate:THF ligase (rxn00690_c0); 5 – 5,10-methenyl-THF 5-hydrolase (rxn01211_c0); 6 – ammonia diffusion (rxn05466_c0); 7 – ATP synthase (rxn10042_c0); 8 – 5,10-methylene-THF:NADP$^+$ oxidoreductase (rxn00907_c0); 9 – ethanol:NAD$^+$ oxidoreductase (rxn00543_c0); 10 – ATP:AMP phosphotransferase (rxn00097_c0); 11 – sulfate transport via proton symport (rxn05651_c0);12 – lactate

transport (rxn05602_c0); 13 – thioredoxin reductase (rxn05289_c0); 14 – acytl-CoA:orthophosphate acetyltransferase (rxn00173_c0); 15 – pyrophosphate phosphohydrolase (rxn00001_c0); 16 – NGAM (ATP phosphohydrolase [protein-secreting]) (rxn00062_c0); 17 – water transport (rxn05319_c0) and CO_2 transport (rxn05467_c0); 18 – hydrogen-sulfide:NADP+ oxidoreductase (rxn00623_c0); * – CO transport (rxn10480_c0), CO dehydrogenase (rxn07189_c0), formate dehydrogenase (rxn00103_c0), CO dehydrogenase/acetyl-CoA synthase (rxn12080_c0), 5-methyl-THF:corrinoid Co-methyltransferase (rxn06149_c0), hydrogen transport (rxn10542_c0), ethanol transport (rxn09683_c0), Rnf complex (M002), acetate transport (rxn05488_c0), NADP-dependent electron-bifurcating [FeFe]-hydrogenase (HytABCDE) (leq000001), Nfn complex (leq000002), formate dehydrogenase/HytABCDE complex (rxn08518_c0), acetaldehyde:ferredoxin oxidoreductase (leq000004); 19 – sulfate adenyltransferase (rxn09240_c0); 20 – sulfate transport (cpd00048_ext_b); 21 – hydrogen sulphide transport (H_2STrans_c0).

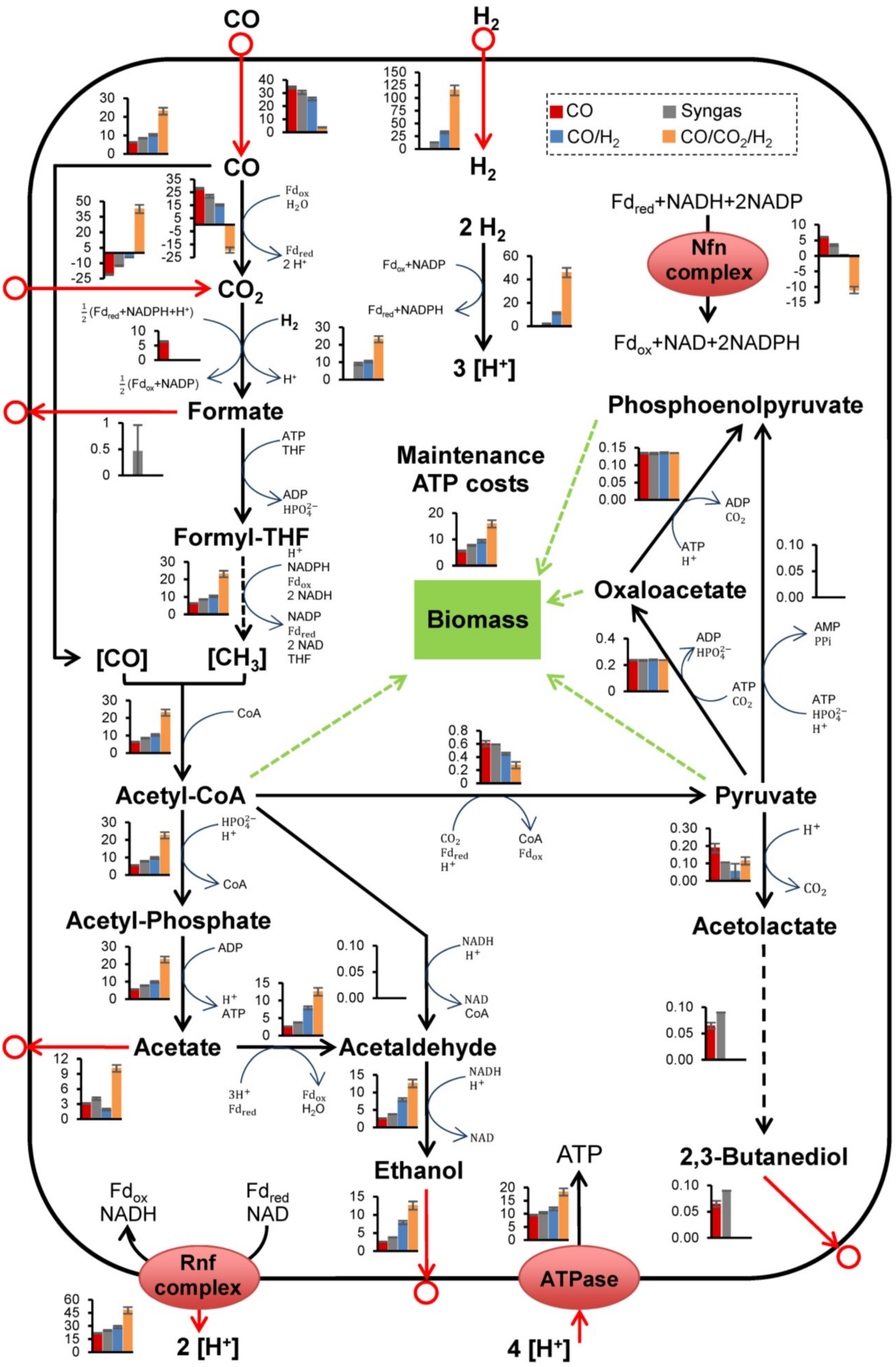
CO
H2
CO
Syngas
CO/H2
CO/CO2/H2
CO
H2
Fdox
H2O
Fdred
2 H+
2 H2
Fdox+NADP
Fdred+NADPH
3 [H+]
Fdred+NADH+2NADP
Nfn complex
Fdox+NAD+2NADPH
CO2
½ (Fdred+NADPH+H+)
H2
½ (Fdox+NADP)
H+
Formate
ATP
THF
ADP
HPO4 2−
Formyl-THF
H+
NADPH
Fdox
2 NADH
NADP
Fdred
2 NAD
THF
[CO]
[CH3]
CoA
Maintenance ATP costs
Biomass
Phosphoenolpyruvate
ADP
CO2
ATP
H+
Oxaloacetate
ADP
HPO4 2−
ATP
CO2
AMP
PPi
ATP
HPO4 2−
H+
Acetyl-CoA
Pyruvate
CO2
Fdred
H+
CoA
Fdox
HPO4 2−
H+
CoA
Acetyl-Phosphate
ADP
H+
ATP
NADH
H+
NAD
CoA
H+
CO2
Acetolactate
Acetate
Acetaldehyde
3H+
Fdred
Fdox
H2O
NADH
H+
NAD
Ethanol
ATP
2,3-Butanediol
Fdox
NADH
Fdred
NAD
Rnf complex
2 [H+]
ATPase
4 [H+]

FIGURE S3 | Intracellular metabolic fluxes in Clostridium autoethanogenum growing on various gas mixes estimated using the metabolic model iCLAU786 and flux balance analysis. Bar charts show specific flux rates (mmol/gDCW/h) from Supplementary Tables S10-11 and represent the average ± standard deviation between biological replicates from SIM: 5-8 (CO), 11-12 (Syngas), 16-19 (CO/H_2), and 20-21 (CO/CO_2/H_2[1]). Results for CO, syngas, and CO/H2 are high biomass condition data from Valgepea et al. (2018), the conditions of these fermentations are summarized in Table S5. Arrows show the direction of calculated fluxes; red arrows denote uptake or secretion, dashed arrows denote a series of reactions. Brackets denote metabolites bound by an enzyme. Refer to Figure S1 & S2 for enzyme involvement, metabolite abbreviations, and reaction IDs, and Supplementary Materials Table S11 for complete flux balance analysis datasets.

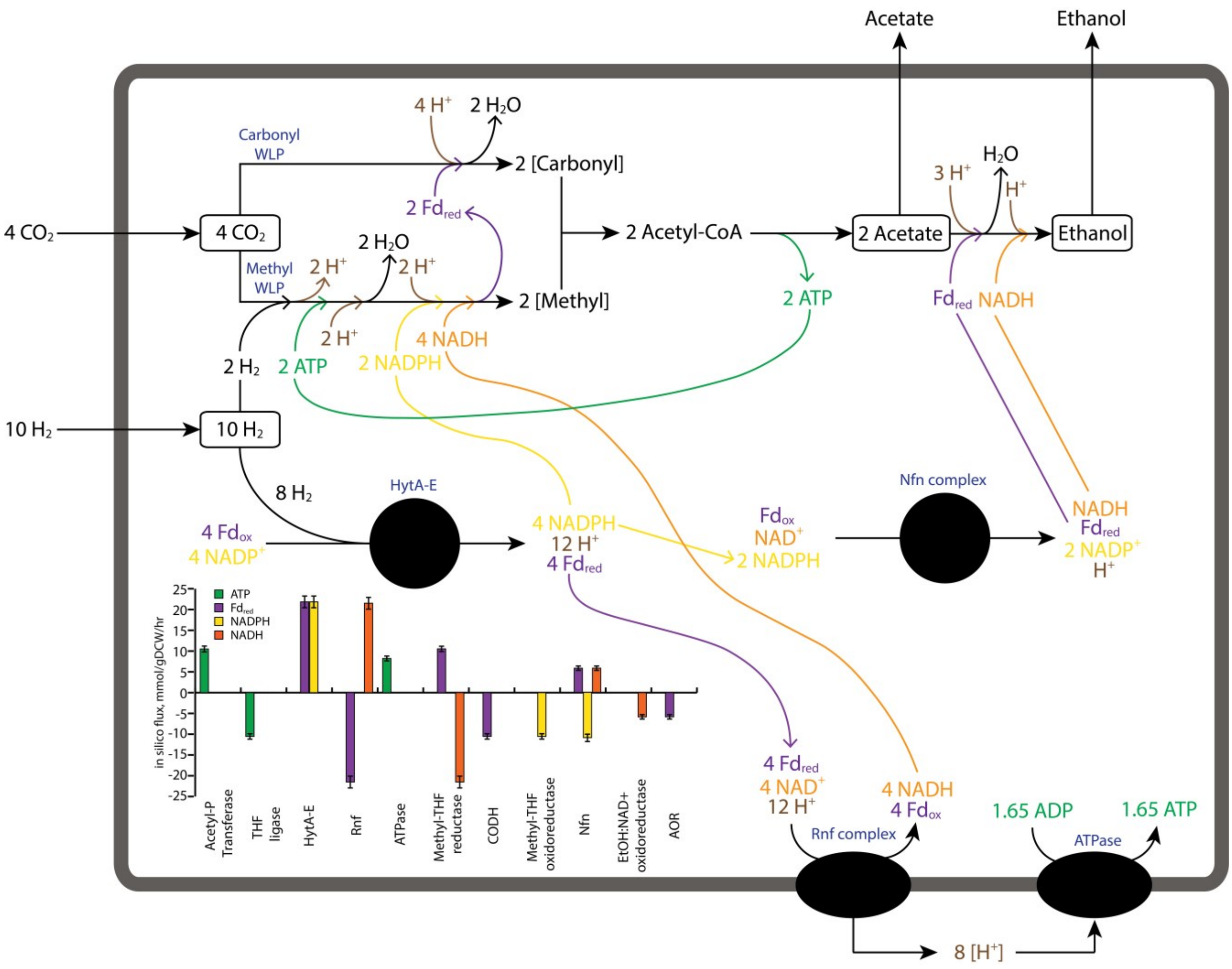

FIGURE S4 | Flux balance analysis of *C. autoethanogenum* fermenting CO_2/H_2 revealed elegant balancing of WLP metabolites and agreement with theoretical stoichiometries from Mock et al. (2015). Estimations of intracellular fluxes are from SIM 22-24, and represented as the average ± standard deviation between biological replicates in the bar chart insert. The approximate overall stoichiometry was $4CO_2 + 10H_2 + 1.65\ ADP + 1.65\ P_i \longrightarrow CH_3COOH + CH_3CH_2OH + 5H_2O + 1.65ATP$; where 1 “stoichiometric reaction mol” (pathways) = ~5 *in silico* flux (mmol/gDCW/hr; bar chart). Refer to Table S7 for flux data displayed here and comparison to CO-supplemented CO_2/H_2 fermentations, Figure S1 & S2 for enzyme involvement, metabolite abbreviations, and reaction IDs, and Supplementary Materials Table S11 for complete flux datasets.

5 References

Adams, M. W. W. (1990). The structure and mechanism of iron-hydrogenases. *BBA - Bioenerg.* 1020, 115–145. doi:10.1016/0005-2728(90)90044-5.

Babicki, S., Arndt, D., Marcu, A., Liang, Y., Grant, J. R., Maciejewski, A., et al. (2016). Heatmapper: web-enabled heat mapping for all. *Nucleic Acids Res.* 44, W147–W153. doi:10.1093/nar/gkw419.

Bengelsdorf, F. R., Beck, M. H., Erz, C., Hoffmeister, S., Karl, M. M., Riegler, P., et al. (2018). Bacterial Anaerobic Synthesis Gas (Syngas) and CO 2 + H 2 Fermentation. *Adv. Appl. Microbiol.* doi:10.1016/bs.aambs.2018.01.002.

Bennett, B., Lemon, B. J., and Peters, J. W. (2000). Reversible carbon monoxide binding and inhibition at the active site of the Fe-only hydrogenase. *Biochemistry* 39, 7455–7460. doi:10.1021/bi992583z.

Bertsch, J., and Müller, V. (2015). Bioenergetic constraints for conversion of syngas to biofuels in acetogenic bacteria. *Biotechnol. Biofuels* 8, 210. doi:10.1186/s13068-015-0393-x.

Bouaifi, M., Hebrard, G., Bastoul, D., and Roustan, M. (2001). A comparative study of gas hold-up, bubble size, interfacial area and mass transfer coefficients in stirred gas-liquid reactors and bubble columns. *Chem. Eng. Process.* 40, 97–111. doi:10.1016/S0255-2701(00)00129-X.

Bürstel, I., Siebert, E., Frielingsdorf, S., Zebger, I., Friedrich, B., and Lenz, O. (2016). CO synthesized from the central one-carbon pool as source for the iron carbonyl in O2-tolerant [NiFe]-hydrogenase. *Proc. Natl. Acad. Sci. U. S. A.* 113, 14722–14726. doi:10.1073/pnas.1614656113.

Ceccaldi, P., Schuchmann, K., Müller, V., and Elliott, S. J. (2017). The Hydrogen Dependent CO_2 reductase: The first completely co tolerant fefe-hydrogenase. *Energy Environ. Sci.* 10, 503–508. doi:10.1039/c6ee02494g.

Charubin, K., and Papoutsakis, E. T. (2019). Direct cell-to-cell exchange of matter in a synthetic Clostridium syntrophy enables CO_2 fixation, superior metabolite yields, and an expanded metabolic space. *Metab. Eng.* 52, 9–19. doi:10.1016/j.ymben.2018.10.006.

Chemistry Reference Reactions. Available at: http://chemistry-reference.com/rxnlist.asp?sort=rxn&language=en.

Chen, J., Daniell, J., Griffin, D., Li, X., and Henson, M. A. (2018). Experimental testing of a spatiotemporal metabolic model for carbon monoxide fermentation with Clostridium autoethanogenum. *Biochem. Eng. J.* 129, 64–73. doi:10.1016/j.bej.2017.10.018.

De Lacey, A. L., Fernández, V. M., Rousset, M., and Cammack, R. (2007). Activation and inactivation of hydrogenase function and the catalytic cycle: Spectroelectrochemical studies. *Chem. Rev.* 107, 4304–4330. doi:10.1021/cr0501947.

Demler, M., and Weuster-Botz, D. (2011). Reaction engineering analysis of hydrogenotrophic production of acetic acid by Acetobacterium woodii. *Biotechnol. Bioeng.* 108, 470–474. doi:10.1002/bit.22935.

Evans, R. M., Brooke, E. J., Wehlin, S. A. M., Nomerotskaia, E., Sargent, F., Carr, S. B., et al. (2016). Mechanism of hydrogen activation by [NiFe] hydrogenases. *Nat. Chem. Biol.* 12, 46–50. doi:10.1038/nchembio.1976.

Fontecilla-Camps, J. C., Volbeda, A., Cavazza, C., and Nicolet, Y. (2007). Structure/function relationships of [NiFe]- and [FeFe]-hydrogenases. *Chem. Rev.* 107, 4273–4303. doi:10.1021/cr050195z.

Igamberdiev, A. U., and Kleczkowski, L. A. (2009). Metabolic systems maintain stable non-equilibrium via thermodynamic buffering. *BioEssays* 31, 1091–1099. doi:10.1002/bies.200900057.

Kantzow, C., and Weuster-Botz, D. (2016). Effects of hydrogen partial pressure on autotrophic growth and product formation of Acetobacterium woodii. *Bioprocess Biosyst. Eng.* 39, 1325–1330. doi:10.1007/s00449-016-1600-2.

Klasson, K. T., Ackerson, M. D., Clausen, E. C., and Gaddy, J. L. (1991). Bioreactor design for synthesis gas fermentations. *Fuel* 70, 605–614. doi:10.1016/0016-2361(91)90174-9.

Klasson, K. T., Ackerson, M. D., Clausen, E. C., and Gaddy, J. L. (1992). Bioconversion of synthesis gas into liquid or gaseous fuels. *Enzyme Microb. Technol.* 14, 602–608. doi:10.1016/0141-0229(92)90033-K.

Lamle, S. E., Albracht, S. P. J., and Armstrong, F. A. (2005). The Mechanism of Activation of a [NiFe]-Hydrogenase by Electrons, Hydrogen, and Carbon Monoxide. *J. Am. Chem. Soc.* 127, 6595–6604. Available at: https://pubs.acs.org/doi/pdf/10.1021/ja0424934.

Lange, H. C., Eman, M., Van Zuijlen, G., Visser, D., Van Dam, J. C., Frank, J., et al. (2001). Improved rapid sampling for in vivo kinetics of intracellular metabolites in Saccharomyces cerevisiae. *Biotechnol. Bioeng.* 75, 406–415. doi:10.1002/bit.10048.

Li, X., Griffin, D., Li, X., and Henson, M. A. (2019). Incorporating hydrodynamics into spatiotemporal metabolic models of bubble column gas fermentation. *Biotechnol. Bioeng.* 116, 28–40. doi:10.1002/bit.26848.

Liew, F. M., Henstra, A. M., Köpke, M., Winzer, K., Simpson, S. D., and Minton, N. P. (2017). Metabolic engineering of Clostridium autoethanogenum for selective alcohol production. *Metab. Eng.* doi:10.1016/j.ymben.2017.01.007.

Liew, F. M., Henstra, A. M., Winzer, K., Köpke, M., Simpson, S. D., and Minton, N. P. (2016). Insights into CO_2 fixation pathway of Clostridium autoethanogenum by targeted mutagenesis. *MBio*. doi:10.1128/mBio.00427-16.

Liu, C., Colón, B., Ziesack, M., Silver, P. A., and Nocera, D. G. (2016). Water splitting-biosynthetic system with CO_2 reduction efficiencies exceeding photosynthesis. *Science (80-.).* 352, 1210–1213. doi:10.1126/science.aaf5039.

Lubitz, W., Ogata, H., Rüdiger, O., and Reijerse, E. (2014). Hydrogenases. *Chem. Rev.* 114, 4081–4148. doi:10.1021/cr4005814.

Lubitz, W., Reijerse, E., and van Gastel, M. (2007). [NiFe] and [FeFe] hydrogenases studied by advanced magnetic resonance techniques. *Chem. Rev.* 107, 4331–4365. doi:10.1021/cr050186q.

Ma, Y., Yu, G., and Li, H. Z. (2005). Note on the mechanism of interfacial mass transfer of absorption processes. *Int. J. Heat Mass Transf.* 48, 3454–3460. doi:10.1016/j.ijheatmasstransfer.2005.03.008.

Marcellin, E., Behrendorff, J. B., Nagaraju, S., DeTissera, S., Segovia, S., Palfreyman, R. W., et al. (2016). Low carbon fuels and commodity chemicals from waste gases – Systematic approach to understand energy metabolism in a model acetogen. *Green Chem.* 18, 3020–3028. doi:10.1039/C5GC02708J.

Mock, J., Zheng, Y., Mueller, A. P., Ly, S., Tran, L., Segovia, S., et al. (2015). Energy conservation associated with ethanol formation from $H_2$and CO_2in Clostridium autoethanogenum involving electron bifurcation. *J. Bacteriol.* doi:10.1128/JB.00399-15.

Molitor, B., Mishra, A., and Angenent, L. T. (2019). Power-to-protein: converting renewable electric power and carbon dioxide into single cell protein with a two-stage bioprocess. *Energy Environ. Sci.* doi:10.1039/C9EE02381J.

Norman, R. O. J., Millat, T., Winzer, K., Minton, N. P., and Hodgman, C. (2018). Progress towards platform chemical production using Clostridium autoethanogenum. *Portl. Press*. doi:10.1042/BST20170259.

Oswald, F., Stoll, I. K., Zwick, M., Herbig, S., Sauer, J., Boukis, N., et al. (2018). Formic acid formation by Clostridium ljungdahlii at elevated pressures of carbon dioxide and hydrogen. *Front. Bioeng. Biotechnol.* 6. doi:10.3389/fbioe.2018.00006.

Pagnier, A., Martin, L., Zeppieri, L., Nicolet, Y., and Fontecilla-Camps, J. C. (2016). CO and CN- syntheses by [FeFe]-hydrogenase maturase HydG are catalytically differentiated events. *Proc. Natl. Acad. Sci. U. S. A.* 113, 104–109. doi:10.1073/pnas.1515842113.

Peters, J. W., Schut, G. J., Boyd, E. S., Mulder, D. W., Shepard, E. M., Broderick, J. B., et al. (2015). [FeFe]- and [NiFe]-hydrogenase diversity, mechanism, and maturation. *Biochim. Biophys. Acta - Mol. Cell Res.* 1853, 1350–1369. doi:10.1016/j.bbamcr.2014.11.021.

Poehlein, A., Cebulla, M., Ilg, M. M., Bengelsdorf, F. R., Schiel-bengelsdorf, B., Whited, G., et al. (2015). The Complete Genome Sequence of *Clostridium aceticum*: a Missing Link between Rnf- and Cytochrome-Containing Autotrophic Acetogens. *MBio* 6, e01168-15. doi:10.1128/mBio.01168-15.Editor.

Qian, H., and Beard, D. A. (2005). Thermodynamics of stoichiometric biochemical networks in living systems far from equilibrium. *Biophys. Chem.* 114, 213–220. doi:https://doi.org/10.1016/j.bpc.2004.12.001.

Quéméner, E. D. Le, and Bouchez, T. (2014). A thermodynamic theory of microbial growth. *ISME J.* 8, 1747–1751. doi:10.1038/ismej.2014.7.

Richter, H., Martin, M. E., and Angenent, L. T. (2013). A two-stage continuous fermentation system for conversion of syngas into ethanol. *Energies* 6, 3987–4000. doi:10.3390/en6083987.

Richter, H., Molitor, B., Diender, M., Sousa, D. Z., and Angenent, L. T. (2016a). A narrow pH range supports butanol, hexanol, and octanol production from syngas in a continuous co-culture of Clostridium ljungdahlii and Clostridium kluyveri with in-line product extraction. *Front. Microbiol.* 7. doi:10.3389/fmicb.2016.01773.

Richter, H., Molitor, B., Wei, H., Chen, W., Aristilde, L., and Angenent, L. T. (2016b). Ethanol production in syngas-fermenting Clostridium ljungdahlii is controlled by thermodynamics rather than by enzyme expression. *Energy Environ. Sci.* doi:10.1039/C6EE01108J.

Taymaz-Nikerel, H., de Mey, M., Ras, C., ten Pierick, A., Seifar, R. M., van Dam, J. C., et al. (2009). Development and application of a differential method for reliable metabolome analysis in Escherichia coli. *Anal. Biochem.* 386, 9–19. doi:10.1016/j.ab.2008.11.018.

Valgepea, K., De Souza Pinto Lemgruber, R., Abdalla, T., Binos, S., Takemori, N., Takemori, A., et al. (2018). H_2 drives metabolic rearrangements in gas-fermenting Clostridium autoethanogenum. *Biotechnol. Biofuels* 11, 55. doi:10.1186/s13068-018-1052-9.

Valgepea, K., de Souza Pinto Lemgruber, R., Meaghan, K., Palfreyman, R. W., Abdalla, T., Heijstra, B. D., et al. (2017). Maintenance of ATP Homeostasis Triggers Metabolic Shifts in Gas-Fermenting Acetogens. *Cell Syst.* 4.

Villadsen, J., Nielsen, J., and Liden, G. (2011). *Bioreaction Engineering Principles*. Third. New York: Springer.

Wang, S., Huang, H., Kahnt, H. H., Mueller, A. P., Köpke, M., and Thauer, R. K. (2013). NADP-Specific electron-bifurcating [FeFe]-hydrogenase in a functional complex with formate dehydrogenase in clostridium autoethanogenum grown on CO. *J. Bacteriol.* 195, 4373–4386. doi:10.1128/JB.00678-13.

Wittkamp, F., Senger, M., Stripp, S. T., and Apfel, U. P. (2018). Hydrogenases: Recent developments and future perspectives. *Chem. Commun.* 54, 5934–5942. doi:10.1039/c8cc01275j.
